# Biomechanical and Functional Improvements Gained by Proximal Tibia Osteotomy Correction of Genu Varum in Patients with Knee Pain

**DOI:** 10.1007/s11420-019-09670-6

**Published:** 2019-03-19

**Authors:** Rachael J. Da Cunha, Andrew P. Kraszewski, Howard J. Hillstrom, Austin T. Fragomen, S. Robert Rozbruch

**Affiliations:** 1 Limb Lengthening and Complex Reconstruction Service, Hospital for Special Surgery, 535 East 70th Street, New York, NY 10021 USA; 2 Kingston Health Sciences Centre, Division of Orthopaedic Surgery, Department of Surgery, Queen’s University, 76 Stuart Street, Kingston, ON K7L 2V7 Canada; 3 Leon Root, MD, Motion Analysis Laboratory, Hospital for Special Surgery, 535 East 70th Street, New York, NY 10021 USA; 4 Weill Cornell Medicine, New York, NY 10065 USA

**Keywords:** gait biomechanics, proximal tibial osteotomy, external fixation, knee pain, osteoarthritis, varus

## Abstract

**Background:**

Mechanical axis malalignment contributes to abnormal forces across the knee joint. Genu varum, or increased medial mechanical axis deviation (MAD), increases force transmission and contact pressures to the medial compartment. With increasing MAD and femoral-tibial mechanical axis angle (MAA), contact forces within the medial or lateral compartment of the knee significantly increase with increasing deformity. This may lead to knee pain, further deformity, and medial compartment degenerative joint disease, which can interfere with participation in sports and diminish quality of life.

**Purposes/Questions:**

We sought to evaluate patients with knee pain with bilateral genu varum and determine the effect of bilateral proximal tibial osteotomies on knee biomechanics, deformity correction, and functional outcomes.

**Methods:**

This was a single-center, prospective study of eight limbs in four patients. Consecutive patients presenting with knee pain and bilateral genu varum originating from the proximal tibia were included. All patients underwent staged bilateral proximal tibial osteotomies with gradual deformity correction with an external fixator. Subjects underwent a three-dimensional (3D) instrumented motion analysis during level walking. A 3D lower extremity model was built and bilateral knee frontal plane kinematics and kinetics during the stance phase of gait were determined. Radiographic analysis was performed including assessment of MAD, MAA, and medial proximal tibial angle (MPTA). Functional outcomes were assessed with the Knee Injury and Osteoarthritis Outcome Score (KOOS), the 36-item Short-Form Survey (SF-36), the Lower Limb Questionnaire (LLQ), and a visual analog scale (VAS) for pain.

**Results:**

The average time in the external fixator for a single limb was 97 days. The average follow-up period was 310 days. All biomechanical outcomes significantly improved, including knee adduction angle (7.8° to 1.8°), knee adduction moments (first peak, − 0.450 to − 0.281 nm/kg, and second peak, − 0.381 to − 0.244 nm/kg), and knee adduction moment impulse (− 0.233 to − 0.150 nm s/kg). There was a significant improvement in MAA, MAD, and MPTA. All patients showed qualitative improvement in mean scores on VAS (11.8 to 0.0), LLQ (77 to 93), KOOS (64 to 84), and SF-36 (71 to 88).

**Conclusion:**

These findings suggest that bilateral proximal tibial osteotomy may be effective in improving knee biomechanics during gait and correcting mechanical alignment in patients with bilateral genu varum. Patients also demonstrated improvement in functional outcome scores. This technique should thus be considered in patients with varus knee osteoarthritis in the setting of genu varum to alleviate symptoms and potentially decrease further clinical deterioration.

## Introduction

Mechanical axis malalignment contributes to abnormal forces across the knee joint. Genu varum, or increased medial mechanical axis deviation (MAD), increases force transmission and contact pressures to the medial compartment [5, 13, 16, 33]. Mootanah et al. examined the effect of malalignment on knee contact forces and found that with increasing MAD and femoral-tibial mechanical axis angle (MAA), contact forces within the medial or lateral compartment of the knee significantly increased with increasing deformity [4]. This may lead to knee pain, further deformity, and medial compartment degenerative joint disease, which can interfere with sport and recreation and activities of daily living, ultimately impacting quality of life [5, 13, 16, 33].

Several studies have demonstrated the effectiveness of a proximal tibial osteotomy in correcting deformity, alleviating pain, and halting the progression of osteoarthritis and subsequent need for knee arthroplasty [1, 4–6, 14, 15, 17, 18, 22, 23, 38]. Satisfactory correction and functional outcomes can be achieved by either acute correction and internal fixation or gradual correction and external fixation with a monolateral or hexapod frame [1, 7–9, 12, 26, 30, 32]. Considerations for performing gradual over acute correction include the severity of deformity or the need for multiplanar correction, such that there is multiplanar deformity or ligament insufficiency requiring sagittal plane correction. Cartilage regeneration has been observed after realignment, even in cases with exposed subchondral bone prior to proximal tibial osteotomy [2, 10, 19, 23, 24].

Several studies have examined clinical outcomes and survivorship of the proximal tibial osteotomy [1, 7–9, 12, 26, 30, 32]. Yasuda et al. demonstrated 5- and 10-year survival to be 95 and 79%, respectively [38], while other long-term studies have demonstrated good to excellent functional results in 75 to 90% at 5 years [1, 7–9, 12, 26, 30, 32]. However, to our knowledge, no studies to date have examined the effect of knee malalignment and subsequent deformity correction on biomechanics during gait. During dynamic loading peak forces are increased, with an average 3.1 times body weight (BW) increase in peak force during walking and 5.4 times BW increase during stair climbing, and thus is important to consider [36]. Measured gait variables of the knee joint are the knee adduction angle (KAA), knee adduction moment (KAM), and knee adduction moment impulse (KAMI). The KAA reflects the frontal alignment between the thigh (hip center to knee center) and shank (knee center to ankle center). The KAM reflects the frontal external rotational forces at the knee and takes into account both subject inertial (limb alignment and geometry) and ground reaction forces (magnitude multiplied by distance to joint center) contributions. KAMI provides a measure of cumulative frontal knee moment loading over time. These biomechanical gait parameters strongly correlate with static and dynamic frontal plane knee alignment and loading [25, 34, 35, 37].

The primary aim of this study was to evaluate patients with bilateral genu varum in the setting of symptomatic early medial compartment knee osteoarthritis and determine the effect of bilateral proximal tibial osteotomies on knee biomechanics, including KAA, KAM, and KAMI. The deformity correction achieved and functional outcomes were also evaluated.

## Methods

This was a single center, prospective study. Institutional review board approval was granted for a pilot study of five subjects, from whom informed consent was obtained. Consecutive patients presenting with knee pain and bilateral genu varum originating from the proximal tibia were identified from March 2012 to March 2013 and indicated for bilateral proximal tibial osteotomies. Exclusion criteria included genu varum not originating from the proximal tibia and unilateral cases. All patients were treated by the two senior authors.

Five patients over the recruitment period met the inclusion criteria and were enrolled in this study. All presented with bilateral proximal tibia vara and underwent staged bilateral proximal tibial medial opening wedge osteotomies with external fixation stabilization. One patient was lost to follow-up. Therefore, there were eight knees in four patients available for final follow-up and analysis. Charts were reviewed to identify patient demographics, including age at the time of surgery, gender, and weight. Radiographic analysis was performed. The severity of osteoarthritis was assessed using the Kellgren and Lawrence grading system [21]. Baseline patient characteristics are summarized in Table 1.

**Table 1:** Patient demographics

	Age (years)	Weight (kg)	Gender		MAA (°)	MAD (mm)	MPTA (°)	K-L Grade
Subject 1	53	86.7	Male	Left	11	35 medial	83	1
				Right	11	42 medial	84	1
Subject 2	37	53.2	Female	Left	5	16 medial	83	1
				Right	5	18 medial	83	2
Subject 3	53	81.8	Male	Left	7	29 medial	81	1
				Right	10	38 medial	82	1
Subject 4	29	84.1	Male	Left	9	33 medial	81	0
				Right	9	38 medial	82	1

*MAA* mechanical axis angle, *MAD* mechanical axis deviation, *MPTA* medial proximal tibia angle, *K-L* Kellgren and Lawrence osteoarthritis severity grade

**Fig. 1:**
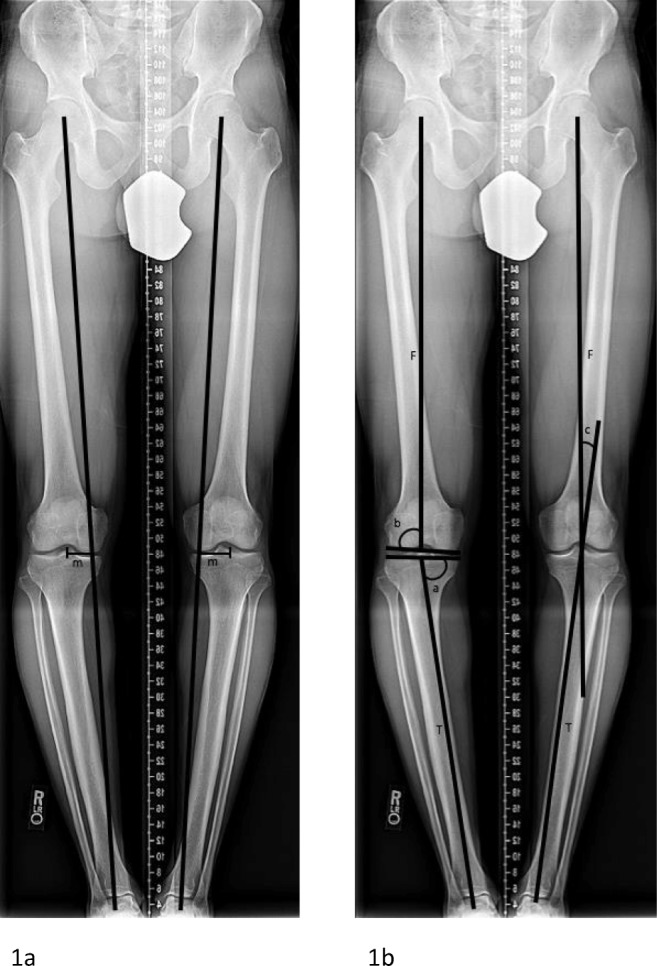
**a**, **b** Radiographic pre-operative assessment of mechanical axis deviation (MAD) (**a**) and medial proximal tibial angle (MPTA), lateral distal femoral angle (LDFA), and mechanical axis angle (MAA) (**b**). *m* MAD, *F* Femur mechanical axis, *T* Tibia mechanical axis, *a* MPTA, *b* LDFA, *c* MAA.

Full-length, standing, bilateral, hip-to-ankle radiographs with the patella centered forward were obtained for all patients. Radiographic analysis included the determination of the mechanical axis line, MAD, MAA, lateral distal femoral angle (LDFA), medial proximal tibial angle (MPTA), and the joint convergence angle (Fig. 1a, b). Genu varum deformity was identified based on increased medial deviation of the MAD (normal, 8 ± 7 mm medial), and proximal tibia vara as the primary source for the deformity was identified as an abnormal MPTA (normal, 87° ± 3°) with a normal LDFA (normal, 88° ± 3°) [3, 28, 29].

Pre-operative planning was then performed for a medial opening wedge proximal tibial osteotomy using either a hexapod external fixator or a monolateral frame. The indication for a hexapod frame was varus greater than 10° or multiplanar deformity [29]. The goal was to restore the mechanical axis to the center of the knee with a straight line from the center of the hip passing between the tibial spines to the floor. A transverse osteotomy was planned at the proximal tibia below the level of the tibial tubercle. The degree of varus correction was then determined at the osteotomy level and correction planned as previously described by Rozbruch et al. [32].

Once indicated for surgery, patients were sent for pre-operative gait analysis. Subjects underwent a three-dimensional (3D) instrumented motion analysis during level walking with a retro-reflective camera system (Motion Analysis Corporation, Santa Rosa, CA, USA) performed both pre-operatively and post-operatively after both external fixators were removed and patients were walking normally. Ground reaction forces were collected with four embedded and synchronized force platforms. Marker and force data were collected at 120 frames per second. Patients were instructed to walk at their comfortable (self-selected) speed until five acceptable trials were recorded. From the trials, a 3D lower-extremity model was built and kinematic and kinetic data were calculated in commercial biomechanical software (Visual3D v6, C-Motion, Inc., Germantown, MD, USA). Biomechanical outcomes included bilateral knee frontal plane kinematics and kinetics during the stance phase of gait.

The anesthetic and surgical protocols were similar for all cases. Regional anesthetic was performed in all cases and included a combination of a neuraxial spinal anesthetic and minimal intravenous sedation. A single dose of pre-incision antibiotics was administered. The patient was positioned supine and a tourniquet was applied to the upper thigh and inflated to 250 mmHg.

In cases where hexapod external fixation was planned, the fibula was first approached to perform a mid-shaft fibular osteotomy. A longitudinal incision was made and the interval between the peroneal muscles and the soleus identified. The fibula surface was identified. An oscillating saw was used to create an oblique bone cut, and a small osteotome was then used to complete the osteotomy.

External fixation with either a hexapod Taylor spatial frame (TSF) (Smith & Nephew, Memphis, TN, USA) or a medial monolateral frame (EBI, Biomet, Warsaw, IN, USA) was utilized. The external fixator was then mounted. In those that received a hexapod frame, proximally a transverse Ilizarov reference wire was first placed from lateral to medial perpendicular to the proximal mechanical axis and a 2/3 ring mounted orthogonal to the proximal tibial mechanical axis. Anterolateral and anteromedial hydroxyapatite-coated half pins were inserted and connected to the proximal ring. Distally, a transverse reference Ilizarov wire was inserted perpendicular to the distal mechanical axis and a full ring mounted orthogonal to the tibial osseous surface at that level. Two multiplanar anteromedial hydroxyapatite half pins were inserted and connected to the distal ring. Mounting parameters were then recorded with the proximal ring set as the reference ring. The position of the center of the proximal ring with respect to the origin in the coronal, sagittal, and axial planes was measured [11]. Hexapod frame struts were then applied and baseline positions were recorded. The struts were then removed in order to perform the tibial osteotomy. The tibial osteotomy site was then identified fluoroscopically just inferior to the tibial tuberosity and based on the pre-operative plan. A 1-cm longitudinal incision was made at this level just medial to the anterior tibial crest. Blunt dissection was carried down to the osseous surface and a periosteal flap raised on each side of the tibial crest. The cortex was predrilled using a 4.8-mm drill in multiple directions along the same plane. A 7-to-10 mm osteotome was used to perform the osteotomy. A complete osteotomy was confirmed clinically by rotating the distal ring externally to the proximal ring, as well as by fluoroscopy. The bone ends were then reduced back to the pre-osteotomy position and the struts reattached in the baseline positions previously recorded.

In the monolateral frame group, only hydroxyapatite pins were used for fixation, with two proximally and two distally. The frame was attached and baseline position noted. The tibial osteotomy was performed with an osteotome after predrilling with a 4.8-mm drill. The lateral cortex was left intact as a hinge, and the fibula was not cut. There was no acute correction performed, and the frame was re-attached in the baseline position previously set.

Post-operatively patients were permitted to be weight bearing as tolerated with crutches. The dressing was removed on post-operative day 2 and pin care initiated, which included daily cleansing with half hydrogen peroxide and half sterile saline solution. Patients were discharged on post-operative day 2 or 3. Patients were placed on 2 weeks of enoxaparin for venous thromboembolism prophylaxis starting on post-operative day 2.

**Fig. 2:**
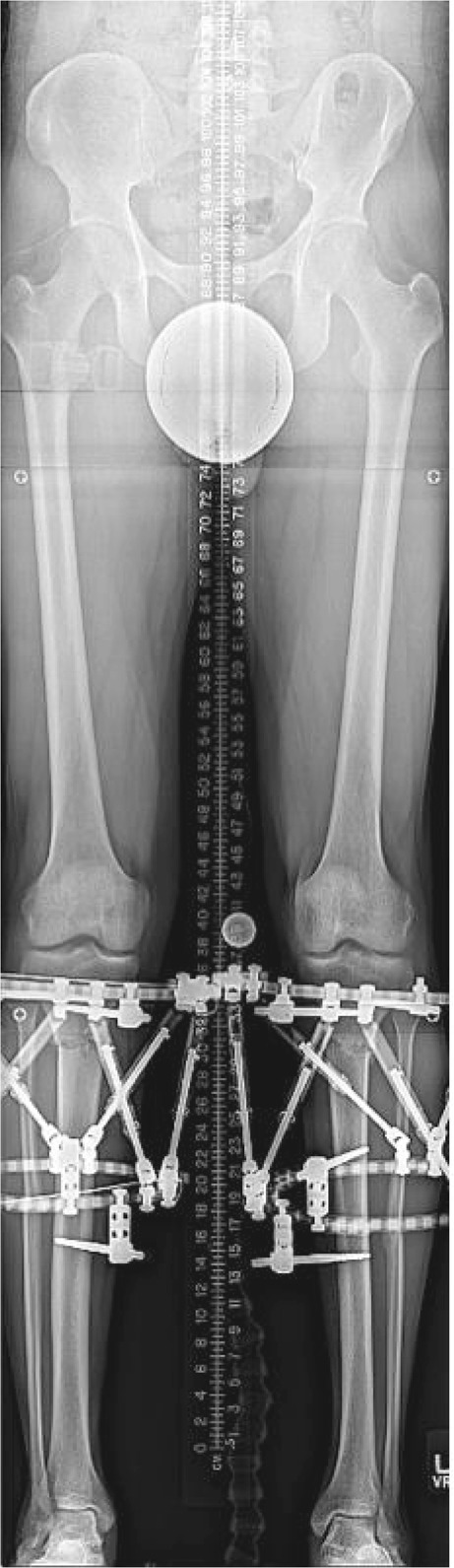
Standing hip-to-ankle radiograph showing neutral alignment once full correction was achieved with bilateral hexapod external fixators in place.

Deformity correction and frame adjustments were initiated at post-operative day 7 with a correction of 1 mm per day in divided doses at the medial cortex. Patients were evaluated every 2 weeks for follow up. Repeat full-length, bilateral, hip-to-ankle standing radiographs with the patella centered forward were obtained once the schedule and all strut adjustments were complete (Fig. 2). The MAA and MAD were measured, and it was determined if any further correction with a residual schedule was required.

Patients returned to the operating room for varus deformity correction of the contralateral side 6 to 8 weeks from the index procedure. At this point, patients were bearing full weight on the operated side. The same pre-operative planning, surgical technique, and post-operative protocol were followed.

The external fixator was removed in the operating room approximately 3 months post-operatively for each side after bridging bone was noted on X-ray. The patient was permitted to be weight bearing as tolerated following external fixator removal.

From the instrumented motion analysis, bilateral knee kinematics and kinetics during level walking were assessed. Eight limbs underwent instrumented motion analysis at an average of 301 days (262 to 370) post-operatively from the final procedure and compared to pre-operative measures. Discrete biomechanical outcomes, taken from gait analysis curves, assessed were stance-averaged KAA during walking and standing phases, first and second peak KAM, and KAMI. Values were compared to normative values determined from ten healthy control subjects who had no knee pain or evidence of deformity or degenerative joint disease.

**Fig. 3:**
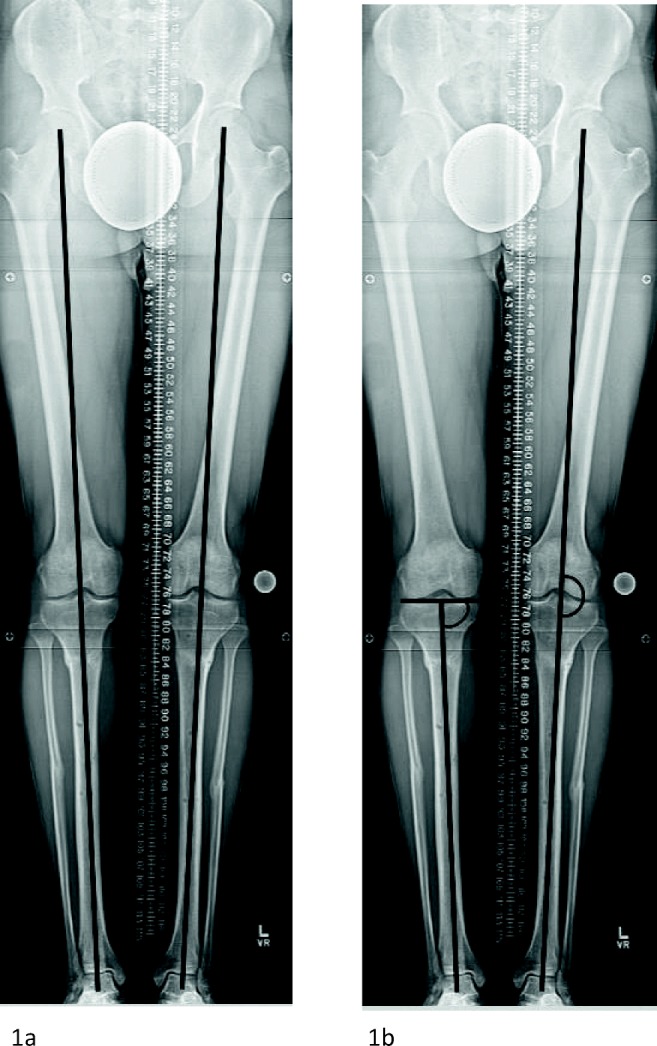
**a**, **b** Standing hip-to-ankle radiograph showing maintenance of correction and neutral alignment once complete consolidation was achieved and bilateral external fixators removed. **a** shows neutral mechanical axis deviation and **b** shows neutral mechanical axis angle and normalization of the medial proximal tibial angle.

Post-operative radiographic analysis was performed, including assessment of MAD, MAA, and MPTA and compared to pre-operative radiographs to assess the deformity correction achieved (Fig. 3a, b).

Functional outcomes were assessed using the Knee Injury and Osteoarthritis Outcome Score (KOOS), the 36-item Short Form Survey (SF-36), and Lower Limb Questionnaire (LLQ). The questionnaires were administered 1 week prior to the motion analysis. Survey scores were obtained at pre- and post-operative time points and standardized from 0 (poor) to 100 (excellent). A visual analog scale (VAS) to measure pain levels was administered pre- and post-operatively and converted to a numeric scale, with 0 indicating no pain and 100 indicating maximum pain.

### Statistical Analysis

Outcomes were compared between pre-operative and post-operative time points with a repeated measures analysis that adjusted for bilateral inter-dependence using generalized estimating equations. Descriptive statistics were reported as the mean ± 1.0 standard deviation (SD). All statistics were done in SPSS (IBM; Armonk, NY, USA).

## Results

Of the five patients initially enrolled in the study, there were four patients, eight limbs, available for follow-up. The average time in the external fixator for a single limb was 97 days (72 to 111).

**Fig. 4:**
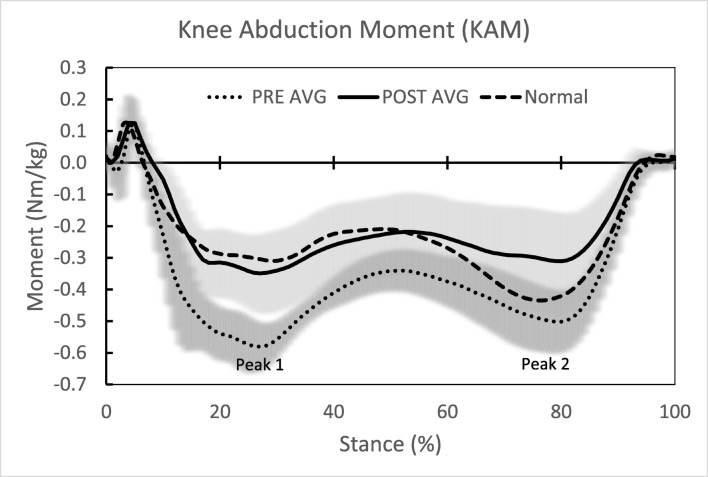
Average pre- and post-operative knee kinetics normalized to stance phase and as compared to controls. Highlighted kinetic outcomes are first and second knee adduction moment peaks.

**Fig. 5:**
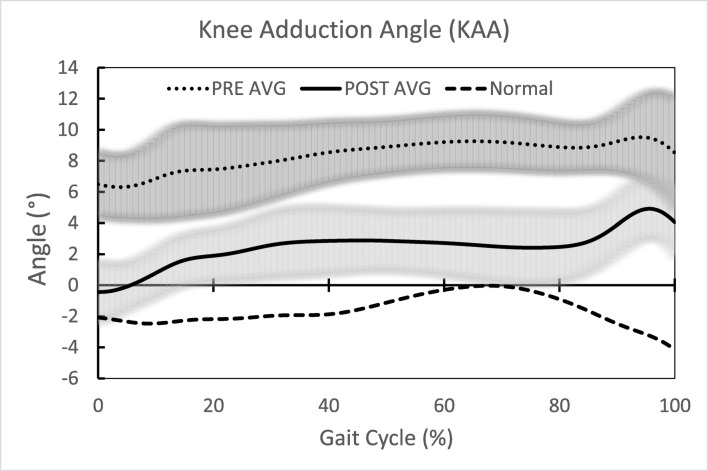
Average pre- and post-operative knee kinematics through the gait cycle and as compared to controls.

All biomechanical outcomes significantly improved pre- to post-operatively, with KAA during walking and standing showing a 54.3 and 76.9% change, respectively; KAM peak 1, peak 2, and KAMI improvement ranged from 35.6 to 37.6% (Table 2). The average pre- and post-operative knee kinetics normalized to stance phase (Fig. 4) and knee kinematics (Fig. 5) through the gait cycle showed significant improvement, as well as normalization, compared to controls.

**Table 2:** Comparison of pre- and post-operative instrumented motion analysis biomechanical outcomes

		KAA Walking (^o^)		KAA Standing (°)		KAM Peak 1 (Nm/kg)		KAM Peak 2 (Nm/kg)		KAMI (Nm∙s/kg)	
		PRE	POST	PRE	POST	PRE	POST	PRE	POST	PRE	POST
Subject 1	Left	9.4	3.8	10.5	3.4	− 0.602	− 0.362	− 0.538	− 0.444	− 0.227	− 0.167
	Right	8.4	7.0	9.2	3.5	− 0.369	− 0.198	− 0.347	− 0.188	− 0.268	− 0.206
Subject 2	Left	5.3	5.1	4.8	2.2	− 0.542	− 0.481	− 0.544	− 0.495	− 0.222	− 0.234
	Right	8.0	4.4	5.5	2.7	− 0.270	− 0.219	− 0.147	− 0.147	− 0.174	− 0.181
Subject 3	Left	9.1	3.0	6.1	1.3	− 0.690	− 0.397	− 0.576	− 0.334	− 0.267	− 0.164
	Right	14.0	5.1	8.4	− 0.1	− 0.298	− 0.161	− 0.216	− 0.100	− 0.260	− 0.140
Subject 4	Left	10.4	0.5	9.8	0.7	− 0.595	− 0.282	− 0.510	− 0.122	− 0.242	− 0.056
	Right	9.2	4.6	8.1	0.7	− 0.236	− 0.148	− 0.171	− 0.117	− 0.202	− 0.081
Average		9.2	4.2	7.8	1.8	− 0.450	− 0.281	− 0.381	− 0.244	− 0.233	− 0.150
Control^a^		− 1.6		− 0.4		− 0.258		− 0.338		− 0.158	
Delta^b^		5.0		6.0		0.169		0.137		0.083	
% Change^c^		54.3		76.9		37.6		35.6		35.6	
*p* value		< 0.001		< 0.001		< 0.001		< 0.001		0.008	

*PRE* preoperative, *POST* postoperative, *KAA* knee adduction angle, *KAM Peak 1* knee adduction moment, *KAM Peak 2* knee adduction moment, *KAMI* knee adduction moment impulse

^a^Control values determined based assessment of 10 healthy subjects with neutral alignment and absence of degenerative joint disease

^b^Absolute change from pre-operative to post-operative

^c^Percent change from pre-operative to post-operative

There was a significant improvement in the radiographic measurements including MAA, MAD, and MPTA when compared pre- to post-operative (Table 3). The final measurements for each were within accepted normal limits [28].

**Table 3:** Radiographic assessment of mechanical axis deviation (MAD), mechanical axis angle (MAA), and medial proximal tibia angle (MPTA) means, pre-operative compared to postoperative

	PRE	POST	*p* value
MAD (mm)	31.1 ± 11.8 medial	5.5 ± 5.6 medial	< 0.001
	(16 to 42)	(0 to 11)	
MAA (°)	8.4 ± 3.1	1.3 ± 1.9	< 0.001
	(5 to 11)	(0 to 4)	
MPTA (°)	82.4 ± 1.3	88.3 ± 1.9	< 0.001
	(81 to 84)	(86 to 90)	

The four patients completed the functional outcome questionnaires at an average of 294 days from the final procedure. Pre- to post-operative improvements were seen in the patients’ VAS pain scores, from a mean of 11.8 to 0.0; LLQ scores, from a mean of 77 to 93; and SF-36 scores, from a mean of 71 to 88 (Table 4). On average, patients showed improvement in KOOS overall, as well as in the individual assessment domains (Table 5). However, one patient showed a decline in KOOS, from an overall mean of 89 to 78. Statistical significance could not be determined for the functional outcomes given the small sample size.

**Table 4:** Quantitative comparison of preoperative and postoperative functional outcome questionnaires

	KOOS		LLQ		SF-36		VAS	
	PRE	POST	PRE	POST	PRE	POST	PRE	POST
Subject 1	38	90	63	100	67	94	23.3 R/37.8 L	0 R/0 L
Subject 2	89	78	91	96	66	88	3.8 R/5.8 L	0 R/0 L
Subject 3	46	73	57	75	54	78	–	–
Subject 4	84	93	97	100	96	93	0 R/0 L	0 R/0L
Average	64	84	77	93	71	88	11.8	0
DELTA^a^	20		16		17		11.8	

*KOOS* Knee Osteoarthritis Outcome Score, *LLQ* Lower Limb Questionnaire, *SF-36* 36-Item Short Form Survey, *VAS* visual analog scale

^a^Absolute change from pre-operative to post-operative

**Table 5:** Quantitative comparison of preoperative and postoperative Knee Osteoarthritis Outcome Score (KOOS) individual domains

	PRE	POST	Delta^a^
KOOS overall	64	84	20
KOOS pain	70	92	22
KOOS other symptoms	83	86	3
KOOS function in daily activities	72	92	20
KOOS function in sport and recreation	56	74	18
KOOS quality of life	39	75	36

^a^Absolute change from pre- to post-operative

## Discussion

The aim of our study was to provide gait analysis confirmation of the value of proximal tibial osteotomy on knee mechanics and gait in patients with genu varum deformity. In this current study, deformity correction was achieved through a proximal tibial osteotomy with gradual correction and external fixation. Final radiographic values for MAA and MAD showed significant improvement and were restored to within accepted normal limits [20, 28]. Associated with this correction were improved knee clinical and functional outcomes scores. The single patient that showed a decline in the KOOS was highly functioning pre-operatively and showed minimal absolute change. The current study demonstrated that there was significant improvement in knee kinetics and kinematics in our cohort of patients following proximal tibial osteotomy correction of bilateral genu varum.

The main limitation of this study is the small sample size of only four subjects. However, this was intended as a pilot study to assess the effect of proximal tibial osteotomy on knee biomechanics and gait in patients with genu varum deformity. Our results suggest an effect on gait biomechanics, laying the groundwork for future research. Our study examined only patients treated with gradual correction and external fixation. The senior authors’ current standard of practice now includes the use of an acute medial opening wedge osteotomy and internal fixation performed for varus of less than 12° and the use of gradual correction with a hexapod frame for varus of greater than 12° or complex multiplanar deformity. This practice shift was made as it was determined that a satisfactory result could be achieved with an acute correction for a less severe deformity and thus not subject a patient to an external fixator if not necessary [31]. Although we do not anticipate a difference in patients treated with acute correction and internal fixation (deformity correction is equivalent), these patients could be included in future works.

Prior studies have examined the effect of coronal malalignment on knee biomechanics. In previous works, a 3D computational knee model was validated using a cadaver model to be accurately predictive of joint contact forces within the knee joint compartments with varying degrees of MAA and MAD. In neutral alignment, contact forces were found to be relatively equal between medial and lateral compartments. With increasing medial MAD and varus MAA, contact forces significantly increased with an MAA and MAD as little as 5° and 15 mm medial, respectively, leading to two times, or 100%, the load transmission through the medial compartment [27]. Our study contributes to the literature by showing that during the gait cycle knee biomechanics are improved following genu varum correction. This confirms that force transmission is decreased during static loading, as well as dynamic loading. Not only was a significant improvement seen pre- to post-operatively, but also normalization compared to healthy controls. Such improvement and decreased force transmission may be, in part, responsible for the significant improvement in pain and functional outcomes demonstrated. This normalization of gait biomechanics with a proximal tibial osteotomy may be protective against further joint degeneration.

In conclusion, bilateral proximal tibial osteotomy was effective in improving knee biomechanics during gait and correcting mechanical alignment in four patients with bilateral genu varum. Patients also demonstrated improvement in functional outcome scores. This technique was shown to be safe and effective and should be considered in patients with mild to moderate varus knee osteoarthritis in the setting of genu varum to alleviate symptoms and potentially decrease arthritis progression and clinical deterioration.
